# Systematic reassessment of reported variants in individuals with suspicion of Alport spectrum disorder reveals a high rate of ambiguous results

**DOI:** 10.1038/s41431-026-02066-1

**Published:** 2026-03-18

**Authors:** Korbinian M. Riedhammer, Patrick Richthammer, Dominik S. Westphal, Jasmina Ćomić, Roman Günthner, Matthias C. Braunisch, Anja K. Büscher, Hanns-Georg Klein, Stefanie Weber, Julia Hoefele

**Affiliations:** 1 Institute of Human Genetics, TUM University Hospital Rechts der Isar, TUM School of Medicine and Health, Munich, Germany; 2 Center for Rare and Genetic Kidney Diseases, Department of Nephrology, TUM University Hospital Rechts der Isar, TUM School of Medicine and Health, Munich, Germany; 3 Division of Nephrology, Department of Pediatrics, Boston Children’s Hospital, Harvard Medical School, Boston, MA USA; 4 Institute of Anatomy, Medical Faculty, University of Bonn, Bonn, Germany; 5 Institute of Human Genetics, University Hospital Salzburg, Paracelsus Medical University, Salzburg, Austria; 6 Department of Pediatrics II, University Hospital Essen, Essen, Germany; 7 Medicover Genetics, Martinsried, Germany; 8 Department of Pediatrics II, University Children’s Hospital, Philipps-University Marburg, Marburg, Germany; 9 Institute of Human Genetics, University Hospital, Ludwig-Maximilians-University, Munich, Germany

**Keywords:** Alport syndrome, Genetics research

## Abstract

“Alport spectrum disorder” describes a phenotypically and genotypically multifaceted disease entity encompassing classic autosomal recessive and X-linked Alport syndrome (AS) but also more heterogenous and typically milder, yet not benign, hematuric phenotypes like autosomal dominant AS, formerly also known as thin basement membrane nephropathy (TBMN). Alport spectrum disorder is associated with disease-causing variants in the type IV collagen genes *COL4A3*, *COL4A4* and *COL4A5*. Variants and genotypes, reported by a genetic diagnostics lab between 2009 and 2014, of 91 index cases with the clinical tentative diagnosis of AS (66/91), TBMN (21/91), or AS/TBMN (not specified further; 4/91), were reassessed based on 2015 ACMG (American College of Medical Genetics and Genomics) criteria and current amendments. 80 different variants, all originally reported as “mutations”, and their genotypes have been reassessed (*COL4A3*: 21/80, *COL4A4*: 15/80, *COL4A5*: 44/80). In 10/80 variants, classification changed from disease-causing to variant of uncertain significance (VUS). 69/91 (76%) index cases included in the analysis could be classified as solved. 22/91 (24%) index cases had an ambiguous result either on variant, genotype, or both variant and genotype level. VUS cases had a significantly more limited phenotype (e.g., isolated microscopic hematuria) compared to non-downgraded cases (e.g., additional extrarenal manifestations). Reassessment of variants/genotypes in this study showed a significant reduction in unequivocal genetic diagnoses highlighting variant and genotype interpretation as a dynamic process. Genetic reports of individuals with suspected Alport spectrum disorder, especially those obtained in the pre-ACMG criteria era, should therefore be critically evaluated.

## Introduction

Disease-causing variants in one of the type IV collagen encoding genes *COL4A3*, *COL4A4*, or *COL4A5* are associated with classic Alport syndrome (AS) and typically milder, while not benign, hematuric phenotypes like autosomal dominant AS (ADAS), formerly known as thin basement membrane nephropathy (TBMN). This was recently summarized as “Alport spectrum disorder” or “Alport kidney diseases” [1–4].

The main phenotype of classic AS is a hematuric progressive kidney disease leading to kidney failure (KF; 90% by age 40 in X-linked AS in males) [5–7]. Associated extrarenal manifestations are hearing impairment and eye anomalies (e.g., anterior lenticonus) [7]. Individuals with milder forms of AS, previously described with the histology-derived term TBMN and now mainly designated as ADAS, feature a hematuric disease with loss of renal function only at a later age and at a lesser percentage (>50 years; about 3% according to population data, about 30% in selected cohorts) [8–10].

Classic AS results from hemizygous disease-causing variants in *COL4A5* (X-linked AS, XLAS) or biallelic disease-causing variants in *COL4A3* and *COL4A4* (autosomal recessive AS, ARAS). As typical for X-linked disorders, females with monoallelic disease-causing variants in *COL4A5* can show a broad phenotypic presentation ranging from clinically asymptomatic to AS, although 95% at least have microscopic hematuria and about 1 in 10 develop KF by age 40 [11].

In individuals with a milder phenotype (ADAS/TBMN), monoallelic disease-causing variants in *COL4A3* and *COL4A4* are often identified [12]. There is much debate if individuals carrying monoallelic disease-causing variants in *COL4A3* and *COL4A4* should be diagnosed with *autosomal dominant* AS [13]. Some object the term ADAS and do not see it as a definite diagnosis, one reason being it does not take into account that an individual with a classic AS phenotype might have a second disease-causing variant in a region not covered by conventional short-read based (exonic) next-generation sequencing (NGS; e.g., intronic variant with splice effect or complex structural rearrangement) [14]. Furthermore, individuals with monoallelic disease-causing variants in *COL4A3*/*COL4A4* have (a) a substantially different disease course than those with classic AS (see above) and (b) these variants have a high prevalence of about 1% in the general population, making the detection of these variants not uncommon in the genomic age of today, questioning the designation of AS in every individual carrying monoallelic disease-causing variants in *COL4A3*/*COL4A4* [10, 15].

On the other side, the term ADAS is commonly used in the literature, one reason stated it is seen as a clear diagnosis which makes clinical follow-up and adherence to therapy easier to achieve [16]. To steer free of this confusion, a gene-centered nomenclature like “Type-IV-collagen-related nephropathy” or a phenotypically broader nomenclature like “Alport spectrum disorder” or “Alport kidney diseases” has been proposed in carriers of disease-causing variants in *COL4A3*-*5* [3, 4, 13, 17]. In the following, the term “Alport spectrum disorder” is used as an umbrella term.

In 2015, the American College of Medical Genetics and Genomics (ACMG) published standards and guidelines for the interpretation of DNA sequence variants, which allowed for a reproducible criteria-based classification of genetic variants into five different categories: benign, likely benign, variant of uncertain significance (VUS), likely pathogenic, and pathogenic [18]. Previous recommendations focused mainly on the criterion that a variant had already been reported as disease-causing in the literature/databases [19]. Nonetheless, based on the growing number of data analysis platforms and major technical advances in human genetics, sequence variant interpretation remains challenging and evolving. Hence, the ACMG criteria from 2015 have been regularly updated since their publication [20, 21]. Therefore, it is important to reassess variants constantly, especially those reported in the pre-ACMG era. Studies from other fields of human genetics have shown that variant interpretation can change in a clinically relevant fashion over time (i.e., from likely pathogenic to VUS) [22]. Furthermore, in Alport spectrum disorder, there is not only the problem of variant interpretation but also genotypic complexities to be considered (see above).

The aim of this study was a reassessment of *COL4A3*-*5* variants reported before the publication of the 2015 ACMG guidelines as disease-causing in individuals with Alport spectrum disorder, both on the variant and on the genotype level.

## Material and methods

### Data collection

Within this study, 80 different variants in *COL4A3* (NM_000091.5), *COL4A4* (NM_000092.5), and *COL4A5* (NM_000495.5) were reviewed as shown below. These variants are mentioned as disease-causing in genetic reports of a cohort of 91 individuals with the clinical tentative diagnosis of Alport spectrum disorder (at the time of the reports documented as either AS [*n* = 66], TBMN [*n* = 21], or AS/TBMN [not specified further; *n* = 4]) between October 2009 and January 2014 and have already been published in Weber et al. [2]. There was no distinction between “likely pathogenic” and “pathogenic” in these pre-ACMG reports. Clinical data was retrieved from this publication. No further clinical data could be collected as individuals did not consent to being re-contacted. The study was performed according to the declaration of Helsinki and approved by the local ethic committees (#73-05, #521/16 S, #24-1030). All included individuals gave written informed consent for retrospective analysis of data.

### Variant reassessment

Variants were thoroughly reassessed based on the 2015 ACMG criteria and amendments [18, 20, 21]. Information about variants were obtained from the following databases (in alphabetical order):

- CADD (https://cadd.gs.washington.edu/snv)
- ClinVar (https://www.ncbi.nlm.nih.gov/clinvar/)
- gnomAD database v2.1.1 (https://gnomad.broadinstitute.org/)
- Human Gene Mutation Database (HGMD®) Professional
- InterVar (http://wintervar.wglab.org/)
- LOVD (https://www.lovd.nl/3.0/search)
- PolyPhen-2 (http://genetics.bwh.harvard.edu/pph2/)
- SIFT (https://sift.bii.a-star.edu.sg/)
- UCSC genome browser (https://genome.ucsc.edu/)
- UniProt (https://www.uniprot.org/)

For in silico prediction of pathogenicity of a variant, a cutoff of >15 for CADD ([23], as recommended on https://cadd.gs.washington.edu/info), a cutoff of >0.85 for PolyPhen-2 [24] and a cutoff of <0.5 for SIFT [25] were used. The PVS1 criterion for predicted loss-of-function (LoF) variants was applied by using the PVS1 decision tree recommended by Tayoun et al. [20]. The PM1 criterion was upgraded to “strong” for glycine substitutions within the collagen triple helix of type IV collagen encoding genes *COL4A3*, *COL4A4* and *COL4A5* as well as for cysteine substitutions within the carboxy NC domain [21, 26].

**Fig. 1:**
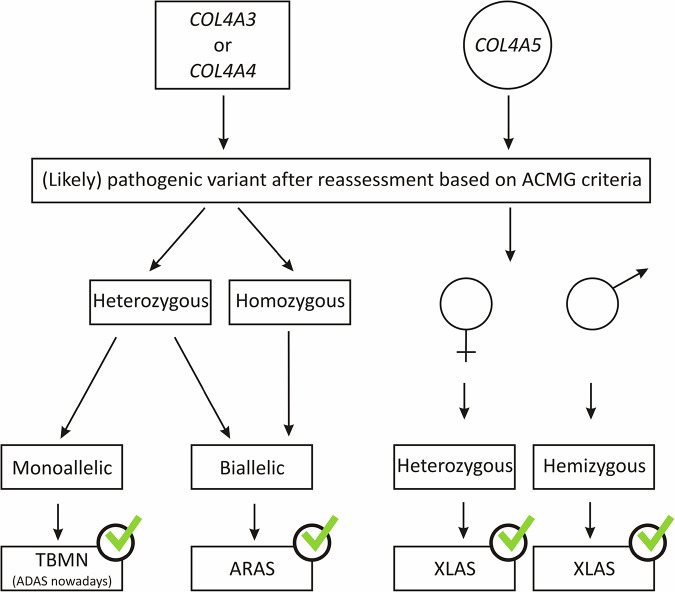
Flow chart with the necessary prerequisites for cases to be designated as solved. ARAS autosomal recessive Alport syndrome, TBMN thin basement membrane nephropathy (as mentioned in the original reports, autosomal dominant Alport syndrome, ADAS, nowadays), XLAS X-linked Alport syndrome.

The workflow for solved cases was defined as described in Fig. 1. Monoallelic (likely) pathogenic variants in *COL4A3*/*4* were considered as causative in individuals with the clinical tentative diagnosis of TBMN (ADAS nowadays), whereas biallelic (likely) pathogenic variants in *COL4A3/4* were considered as causative in individuals with both TBMN and AS (compatible with ARAS). For *COL4A5*, (likely) pathogenic variants were considered as causative in female (heterozygous) and male individuals (hemizygous) with both TBMN and AS (compatible with XLAS).

### Statistics

Statistical analyses were performed with SPSS® Statistics 23 (IBM®, Ehningen, Germany). A significance level (alpha) of 0.05 was chosen.

## Results

### Variant classification after reassessment

**Fig. 2:**
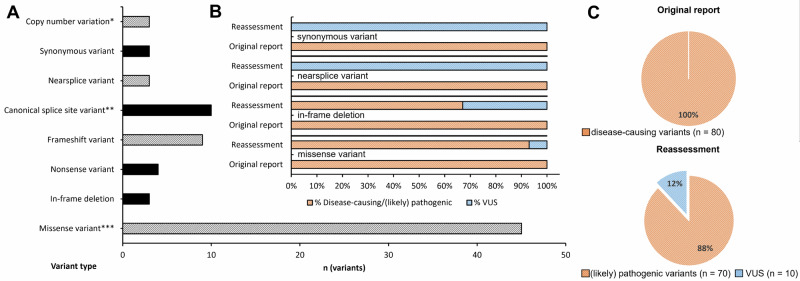
Overview of reassessed variants. **A** Number of variants according to the type of variant. **B** Downgraded variants after reassessment as percentages of respective variant type (no nonsense, frameshift, or canonical splice site variants were downgraded). Ten variants (three missense, three nearsplice, three synonymous variants, one in-frame deletion), which all have been classified as “mutations” (no distinction in likely pathogenic or pathogenic in the pre-ACMG era) in the original report (orange bars), were classified as variants of uncertain significance (VUS) after reassessment (blue bars). **C** Consistently, all variants were classified as disease-causing in the original report, 70/80 (88%) after reevaluation. *, copy number variations were two multi-exon duplications and one single-exon deletion. **, one canonical splice site variant was a 7 base pair deletion involving the canonical splice site. ***, one missense variant led to a start loss. See Supplementary Table for further details on variants.

In total, 80 variants of 91 index cases (*COL4A3* *n* = 17, *COL4A4* *n* = 20, *COL4A5* *n* = 54 cases) and their genotypes have been reassessed (*COL4A3*: 21/80, *COL4A4*: 15/80, *COL4A5*: 44/80). After reassessment of the variants, based on ACMG criteria and amendments, 10 variants were downgraded from disease-causing to VUS, with the result that, in the end, 70 variants were classified as (likely) pathogenic (*COL4A3*: 18 [likely] pathogenic, 3 VUS; *COL4A4*: 14 [likely] pathogenic, 1 VUS; *COL4A5*: 38 [likely] pathogenic, 6 VUS; Fig. 2, Table 1 + 2).

**Table 1:** Distribution of variants in this study.

Gene	Total *n* = 80	Unchanged (likely) pathogenic *n* = 70	Reclassified VUS *n* = 10
*COL4A3*	26% (21/80)	26% (18/70)	30% (3/10)
*COL4A4*	19% (15/80)	20% (14/70)	10% (1/10)
*COL4A5*	55% (44/80)	54% (38/70)	60% (6/10)

**Table 2:** Variants in which the classification changed from disease-causing to VUS after reassessment according to ACMG and amendments.

Gene chromosomal position (hg19)	Variant	Predicted functional consequence	Applied ACMG criteria
*COL4A3* NC_000002.11:g.228153963A>G	c.2979A>G p.(=)	Synonymous variant	PM2
*COL4A3* NC_000002.11:g.228173711_228173713delCAT	c.4559_4561del p.(Ser1520del)	In-frame deletion	PM1 PM2 PM4 (supporting)
*COL4A3* NC_00002.11:g.228131783C>T	c.1483C>T p.(His495Tyr)	Missense variant	PM2 PP3
*COL4A4* NC_000002.11:g.227946893C>G	c.1634G>C p.(Gly545Ala)	Missense variant	PM1 (strong) PP3 BS1
*COL4A5* NC_000023.10:g.107683440A>C	c.81+4A>C p.(?)	Nearsplice variant	PM2
*COL4A5* NC_000023.10:g.107812051G>A	c.384G>A p.(=)	Synonymous variant	PM2 PP3
*COL4A5* NC_000023.10:g.107823977T>G	c.891+9T>G p.(?)	Nearsplice variant	PM2
*COL4A5* NC_000023.10:g.107845218A>G	c.2145A>G p.(=)	Synonymous variant	PM2 PP3
*COL4A5* NC_000023.10:g.107849958T>A	c.2245-14T>A p.(?)	Nearsplice variant	PM2 PP3
*COL4A5* NC_000023.10:g.107938141C>T	c.4793C>T p.(Ser1598Phe)	Missense variant	PM1 PM2 PP3

Transcripts: *COL4A3*, NM_000091.5; *COL4A4*, NM_000092.5; *COL4A5*, NM_000495.5.

*VUS* variant of uncertain significance.

3/10 (30%) VUS were missense variants, 3/10 (30%) were nearsplice variants, 3/10 (30%) were synonymous variants, and 1/10 (10%) was an in-frame deletion (Table 2). No (putative) LoF variants (nonsense, frameshift, canonical splice site variants) or single-/multi-exon deletions/duplications were downgraded to VUS.

### Case classification after reassessment

**Fig. 3:**
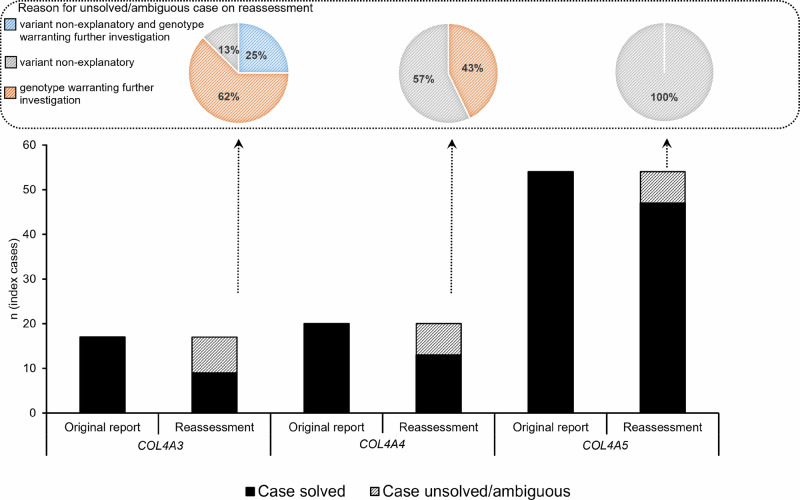
Solved and unsolved/ambiguous cases as described in the original reports and after reassessment. The variants are divided into the different type IV collagen encoding genes *COL4A3, COL4A4* and *COL4A5*. Upper part: More detailed breakdown of the reason for an unsolved/ambiguous case.

69/91 (76%) index cases could be classified as “solved” on variant and genotype level (all 91 cases were classified as solved in the original genetic reports, *p* < 0.00001, Fisher’s exact test). 22/91 (24%) were classified as ambiguous cases either due to inconclusive results on variant (12/22; 55%; clearly unsolved), on genotype (8/22; 36%; definite diagnosis pending, further investigation warranted) or both variant and genotype level (2/22; 9%; clearly unsolved). For X-chromosomal gene *COL4A5*, cases were only unsolved on variant level, while for the autosomal genes *COL4A3*/*COL4A4*, cases were unsolved on variant level or ambiguous on genotype level (or both). A detailed overview of these cases is given in Fig. 3, Tables 3, and 4, respectively.

**Table 3:** Overview of ambiguous cases after reassessment according to 2015 ACMG criteria and amendments (see also Table 4 for further information).

Gene	Ambiguous cases *n* = 22	Variant non-explanatory *n* = 12	Genotype warranting further investigation *n* = 8	Variant non-explanatory and genotype warranting further investigation *n *= 2
*COL4A3* (*n* = 17 cases total)	36% (8/22)	8% (1/12)	63% (5/8)	100% (2/2)
*COL4A4* (*n* = 20 cases total)	32% (7/22)	33% (4/12)	37% (3/8)	0% (0/2)
*COL4A5* (*n* = 54 cases total)	32% (7/22)	58% (7/12)	0% (0/8)	0% (0/2)

**Table 4:** Clinical and genetic characteristics of index cases with ambiguous results (unsolved on variant level/definite diagnosis pending on genotype level) after reassessment according to 2015 ACMG criteria and amendments. Phenotypic information is taken from Weber et al. [2].

Index Case (sex)	Origin	Age at clinical assessment and molecular genetic testing (years)	Microscopic hematuria	Proteinuria	Kidney failure	Hearing impairment	Ocular abnormalities	Reported tentative clinical diagnosis	Gene	Chromosomal position (hg19)	Nucleotide change	Amino acid change	Zygosity	Inhe-ritance	gnomAD v.2.1.1 MAF/occurrence (CNVs)	Applied ACMG criteria	ACMG rating	Ambiguous result due to variant and/or genotype
**13040098 (m)**	Germany	35	yes	yes	yes	yes	yes	AS	*COL4A3*	NC_000002.11:g.228029444T>A	c.2T>A	p.(Met1Lys)	het	n.d.	not listed	PVS1 PM2 PM5 PP3	pathogenic	genotype^a^
										NC_000002.11:g.228145304_228145310delGAGGTAC	c.2372_2374+4delGAGGTAC	p.(?)	het	n.d.	not listed	PVS1 PM2	likely pathogenic	
**13330344 (f)**	Germany	6	yes	no	no	no	no	AS	*COL4A3*	NC_000002.11:g.228109073G>T	c.272G>T	p.(Gly91Val)	het	n.d.	not listed	PM1 (strong) PM2 PM5 PP3	likely pathogenic	genotype
**12200453 (f)**	Spain	35	yes	yes	no	yes	no	AS	*COL4A3*	NC_000002.11:g.228120751G>A	c.898G>A	p.(Gly300Arg)	het	mother	0.00002005	PM1 (strong) PM2 PP3 PS4 (moderate)	likely pathogenic	genotype
**12180174 (f)**	Germany	11	yes	no	no	no	no	TBMN	*COL4A3*	NC_000002.11:g.228131783C>T	c.1483C>T	p.(His495Tyr)	het	n.d.	0.0007191	PM2 PP3	VUS	variant
**10400099 (m)**	Austria	25	n.d.	n.d.	yes	yes	n.d.	AS	*COL4A3*	NC_000002.11:g.228176554C>T	c.4981C>T	p.(Arg1661Cys)	het	n.d.	0.0003596	PM1 PM2 PP3 PM5 PS4 (moderate)	likely pathogenic	genotype^a^
										NC_000002.11:g.228147081G>A	c.2489G>A	p.(Gly830Asp)			not listed	PM1 (strong) PM2 PP3	likely pathogenic	
**13200065 (f)**	Germany	8	yes	yes	no	no	no	AS	*COL4A3*	NC_000002.11:g.228153963A>G	c.2979A>G	p.(=)	het	n.d.	not listed	PM2	VUS	variant and genotype
										NC_000002.11:g.228172594T>C	c.4421T>C	p.(Leu1474Pro)			0.002664	PM1 PM2 PP3 PS4 (moderate)	likely pathogenic	
**10480239 (m)**	Germany	38	yes	yes	n.d.	no	yes	AS	*COL4A3*	NC_000002.11:g.228169782G>A	c.4235G>A	p.(Gly1412Asp)	het	n.d.	not listed	PM1 (strong) PM2 PP3 PM5	likely pathogenic	genotype
**10360333 (f)**	Germany	9	yes	yes	no	no	no	AS	*COL4A3*	NC_000002.11:g.228173711_228173713delCAT	c.4559_4561del	p.(Ser1520del)	het	n.d.	not listed	PM1 PM2 PM4 (supporting)	VUS	variant and genotype
										NC_000002.11:g.228172453G>A	c.4280G>A	p.(Gly1427Asp)			not listed	PM1 (strong) PM2 PP3	likely pathogenic	
**12250266 (f)**	Germany	32	yes	yes	no	no	no	AS	*COL4A4*	NC_000002.11:g.227946893C>G	c.1634G>C	p.(Gly545Ala)	het	father	0.02739	PM1 (strong) PP3 BS1	VUS	variant
**13240632 (m)**	Germany	25	yes	yes	no	no	no	TBMN	*COL4A4*	NC_000002.11:g.227946893C>G	c.1634G>C	p.(Gly545Ala)	het	n.d.	0.02739	PM1 (strong) PP3 BS1	VUS	variant
**13380489 (f)**	Germany	23	n.d.	n.d.	no	yes	no	AS	*COL4A4*	NC_000002.11:g.227946893C>G	c.1634G>C	p.(Gly545Ala)	het	mother	0.02739	PM1 (strong) PP3 BS1	VUS	variant
**13290254 (m)**	Germany	5	yes	no	no	no	no	TBMN	*COL4A4*	NC_000002.11:g.227946893C>G	c.1634G>C	p.(Gly545Ala)	het	father	0.02739	PM1 (strong) PP3 BS1	VUS	variant
**11060199 (m)**	Germany	48	yes	yes	yes	n.d.	n.d.	AS	*COL4A4*	NC_000002.11:g.227920687C>T	c.2690G>A	p.(Gly897Glu)	het	mother	not listed	PM1 (strong) PM2 PP3 PS4 (moderate)	likely pathogenic	genotype
**2290513 (m)**	Germany	8	yes	no	no	no	no	AS	*COL4A4*	NC_000002.11:g.227915821C>T	c.3022G>A	p.(Gly1008Arg)	het	father	0.00003204	PM1 (strong) PM2 PP3	likely pathogenic	genotype
**11050418 (f)**	Germany	41	no	yes	yes	yes	no	AS	*COL4A4*	NC_000002.11:g.227896736C>A	c.3742G>T	p.(Gly1248*)	het	mother	not listed	PVS1 PM2 PP3	pathogenic	genotype
**13380435 (m)**	Germany	5	yes	no	no	no	no	AS	*COL4A5*	NC_000023.10:g.107683440A>C	c.81+4A>C	p.(?)	hemi	mother	not listed	PM2	VUS	variant
**12290515 (m)**	Germany	7	yes	no	no	no	no	AS	*COL4A5*	NC_000023.10:g.107812051G>A	c.384G>A	p.(=)	hemi	mother	not listed	PM2 PP3	VUS	variant
**11200302 (m)**	Germany	16	yes	no	no	no	no	AS	*COL4A5*	NC_000023.10:g.107823977T>G	c.891+9T>G	p.(?)	hemi	mother	not listed	PM2	VUS	variant
**12290425 (f)**	Germany	2	yes	no	no	no	no	AS	*COL4A5*	NC_000023.10:g.107845218A>G	c.2145A>G	p.(=)	het	mother	not listed	PM2 PP3	VUS	variant
**13100284 (f)**	Germany	15	yes	yes	no	no	no	AS	*COL4A5*	NC_000023.10:g.107849958T>A	c.2245-14T>A	p.(?)	het	n.d.	not listed	PM2 PP3	VUS	variant
**10400018 (m)**	Germany	14	yes	yes	no	no	no	AS	*COL4A5*	NC_000023.10:g.107938141C>T	c.4793C>T	p.(Ser1598Phe)	hemi	mother	not listed	PM1 PM2 PP3	VUS	variant
**11120208 (m)**	Türkiye	5	yes	yes	no	n.d.	n.d.	AS	*COL4A5*	NC_000023.10:g.107938141C>T	c.4793C>T	p.(Ser1598Phe)	hemi	mother	not listed	PM1 PM2 PP3	VUS	variant

Transcripts: *COL4A3*, NM_000091.5; *COL4A4*, NM_000092.5; *COL4A5*, NM_000495.5.

*AS* Alport syndrome, *hemi* hemizygous, *het* heterozygous, *n.d. *not determined, *TBMN* thin basement membrane nephropathy (autosomal dominant Alport syndrome, *ADAS*, nowadays), *VUS* variant of uncertain significance.

^a^Compound-heterozygosity not determined.

### Clinical characteristics of the total cohort and index cases with ambiguous results after reassessment

All index cases with respective variants and clinical information at time of genetic testing are listed in the Supplementary Table. All ambiguous cases are additionally listed in Table 4.

In cases with only microscopic hematuria (*n* = 23/91, 25%), median age at genetic testing was 7 years (range 2–41 years) vs. 28 years (range 3–44 years) in cases that did not have reported microscopic hematuria (*n* = 16/91, 18%; *p* < 0.001, Mann-Whitney *U* Test). This is also true for the ambiguous cases, where *n* = 8/22 (36%) only had microscopic hematuria and had genetic testing at a median age of 7 years (range 2–16 years) vs. 25 years (range 23–41 years) in cases which did not have reported microscopic hematuria (*n* = 3/22, 14%).

Along these lines, for *COL4A5* cases (*n* = 54/91, 59%), where genotypic aspects do not play a role for genetic diagnosis (in contrast to autosomal genes *COL4A3*/*COL4A4*, variants in *COL4A5* are diagnostic irrespective of clinical tentative diagnosis in both females and males), individuals with variants classified as VUS (*n* = 7/54, 13%; Table 4) had a median age at genetic testing of 7 years (range 2–16 years) and a limited phenotype only comprising microscopic hematuria with or without proteinuria (no KF and/or eye/ear extrarenal features). However, for solved *COL4A5* cases (*n* = 47/54, 87%; Supplementary Table), median age was 16 years (range 2–62 years; *p* = 0.04, Mann-Whitney *U* Test) and *n* = 21/47 (45%) of these individuals have at least one phenotype of KF or eye/ear extrarenal manifestations (*p* = 0.04, Fisher’s exact test).

None of the three ambiguous cases with the clinical tentative diagnosis of TBMN (ADAS nowadays) had KF and/or extrarenal manifestations (hearing impairment/ocular abnormalities). If one or more of these features (KF, eye/ear extrarenal manifestations) were present, cases had the clinical tentative diagnosis of AS (even without microscopic hematuria; case 13380489, for example). This is also true for the total cohort of *n* = 91 cases.

One non-explanatory variant stood out: Missense variant NM_000092.5:c.1634G>C, p.(Gly545Ala) in *COL4A4*, a now known benign variant (ClinVar Variation ID: 255015; allele frequency of 3.7% in Non-Finnish Europeans in gnomAD v.2.1.1) was reported in four independent individuals. One (13380489), a 23-year-old female with the clinical tentative diagnosis of AS, only had hearing impairment at the time of genetic testing (microscopic hematuria/proteinuria were not determined). The other three (individuals 12250266, 13240632, and 13290254) had microscopic hematuria. Two of these three also had proteinuria (12250266, 32-year-old female, and 13240632, 25-year-old male), one (the female) had the tentative diagnosis of AS, the other (the male) had the tentative diagnosis of TBMN (Table 4).

Finally, cases 13040098 and 10400099 were ambiguous on genotype level although two (likely) pathogenic variants in the autosomal gene *COL4A3* were identified in each case. This was due to the lack of segregation of the variants in the parents to confirm a biallelic state.

## Discussion

The diagnosis of hereditary kidney disease, and any other hereditary disease, must be based on transparent and reproducible principles. With the criteria published in 2015, ACMG introduced standards on sequence variant interpretation in monogenic disorders. It has since been shown that variant classification is not set in stone, for example in hereditary pediatric cardiomyopathy cases, and precise and recurrent variant interpretation is warranted, as also illustrated in a study reassessing variants of individuals included in the EARLY PRO-TECT Alport trial [27, 28].

In Alport spectrum disorder, involving autosomal and X-linked inheritance patterns and profound phenotypic variability, sequence alterations in the associated genes *COL4A3*, *COL4A4*, and *COL4A5* are not only to be meticulously interpreted on the variant but also genotype level.

Individuals with a heterozygous disease-causing variant in *COL4A3* or *COL4A4* have an increased risk of developing KF compared to the general population (at least about 3% at age 60 vs. about 0.1% in the general population) [10, 29–31]. Correct interpretation of identified variants is crucial to provide these individuals with the appropriate recommendations, including further diagnostics, monitoring and treatment [32]. This is also important for relatives of the index individual who may opt for predictive testing. The result and its possible misinterpretation can have substantial impact on their entire life (e.g., reduction in quality of life, reproductive choices, disease management).

Reassessment of variants and genotypes in this study showed a significant shift from unequivocal genetic diagnoses (*n* = 69/91 vs. *n* = 91/91, *p* < 0.00001) to genetically unsolved cases (inconclusive result on variant or variant and genotype level) or cases, in which the definite diagnosis was pending and warranted further investigation (inconclusive result on genotype level). On variant reassessment, the predominant change from (likely) pathogenic to VUS was seen for missense, nearsplice, and synonymous variants (each *n* = 3/10 [30%].; Fig. 2B). In turn, (likely) pathogenic putative loss-of-function variants like frameshift, nonsense and canonical splice site variants, did not alter to VUS at all (Table 2). This is not surprising, as evidence for pathogenicity is usually stronger for LoF variants in comparison to missense variants, especially if the latter are not involving glycine residues [21, 33]. In turn, it is noteworthy that nearsplice and synonymous variants were reported as disease-causing without further functional evidence (i.e., effect on splicing). Maybe, this was merely due to the rarity of the variants (Table 4) and at the time of the report, in the pre-ACMG era, this was enough to classify these as “mutations.”

More than a third of the ambiguous cases (*n* = 8/22, 36%) could not be conclusively solved on genotype level (AS as clinical tentative diagnosis but only a heterozygous disease-causing variant in *COL4A3*/*COL4A4*), i.e., the definite diagnosis was still pending and follow-up investigation like further clinical phenotyping and segregation analyses were warranted (Fig. 3 and Table 4). Many laboratories would diagnose these cases as ADAS today, not considering the ambiguity of this result and the need to thoroughly reverse phenotype these individuals and maybe engage in additional diagnostics, e.g., for intronic variants, before labeling a case as solved in a monogenic “*Alport syndrome*” context (see paragraphs above and “Introduction”) [34]. This again underlines that genetic tests in Alport spectrum disorder are to be meticulously interpreted not only on variant but also genotype level. For variants in *COL4A5*, we did not experience genotype interpretation discrepancies, as we considered heterozygous female carriers of disease-causing variants, due to the variable expressivity of XLAS in heterozygous females, as genetically solved irrespective if the diagnosis was TBMN or AS on the genetic report (Fig. 1 and Fig. 3).

We also reevaluated the clinical data of the individuals as published in Weber et al. in light of the reassessment on variant and genotype level [2]. Interestingly, if microscopic hematuria was present in an individual at the time of genetic testing, the median age at genetic testing was significantly lower than if no microscopic hematuria was present (7 vs. 28 years, *p* < 0.001). This underscores that (microscopic) hematuria is an early guiding feature of Alport spectrum disorder which prompts genetic testing, as also recognized in the 2024 Alport syndrome guideline on behalf of ERKNet, ERA and ESPN [35].

Furthermore, for *COL4A5*, solved cases had an older median age at diagnoses than those in which variants had been downgraded to VUS (7 vs. 16 years, *p* = 0.04). Additionally, VUS cases only exhibited a limited phenotype of microscopic hematuria with or without proteinuria, while 47% of solved cases had at least one phenotype of KF or extrarenal manifestations (*p* = 0.04). This suggests that, in terms of genetic testing, jumping at limited phenotypes, albeit hallmark features, at an early age (i.e., isolated microscopic hematuria) could lead to more ambiguous results due to lower pre-test probabilities in comparison to cases in which there is a more severe phenotype (e.g., microscopic hematuria with KF and extrarenal manifestations).

However, this possibility should not limit diagnostics, as half of the *COL4A5* cases solved still had no KF or extrarenal phenotype. It must be stressed that early diagnosis and treatment is still key, and no physician should wait until more severe features arise before initiating genetic testing if suspicion is high for Alport spectrum disorder [35]. Nonetheless, our data indicates that limited phenotypes could then lead to more ambiguous results on variant level. It is paramount to evaluate these cases in interdisciplinary (nephrogenetic) teams and boards.

Along these lines, it is not surprising that TBMN was only considered as clinical tentative diagnosis in those individuals with a limited phenotype of microscopic hematuria and not if KF and/or extrarenal manifestations were present. It is well known that heterozygous carriers of (likely) pathogenic variants in *COL4A3*/*COL4A4*, which are identified in individuals with TBMN (ADAS nowadays), typically do not feature KF (or only at >50 years of age) and eye/ear abnormalities [10].

The repeatedly identified missense variant NM_000092.5:c.1634G>C, p.(Gly545Ala) in *COL4A4* illustrates how important sequencing databases of the general population, like gnomAD, are for variant interpretation (gnomAD’s predecessor, ExAC, was made publicly available in 2014, after the original genetic reports evaluated in this study) [36]. Although being a glycine change in the triple-helix domain, p.(Gly545Ala) has an allele frequency of 3.7% in Non-Finnish Europeans (gnomAD v.2.1.1) and is a known benign variant (ClinVar Variation ID: 255015). Fittingly, none of the four individuals carrying this variant in a heterozygous state had developed KF or had extrarenal features (median age at genetic testing 24 years, range 5–32 years).

A major limitation of this study is that no follow-up clinical data was available for the individuals but only genetic reports with the clinical tentative diagnosis of AS or TBMN and the published clinical data from Weber et al. [2]. However, this condition reflects the daily routine of a genetic diagnostic laboratory, which is often only provided with rudimentary clinical information or tentative diagnoses (which, of course, should not be the standard of care). Furthermore, our approach on genotype evaluation is very strict. Nonetheless, the intention of this study was to highlight the complexity of Alport spectrum disorder genetics and phenotypes and especially instill skepticism in reports from the pre-ACMG era and of heterozygous carriers of disease-causing variants in *COL4A3*/*COL4A4*.

Of note, heterozygous disease-causing variants in *COL4A3*/*COL4A4* are estimated to have a prevalence of about 1:100 in the general population [15]. Given the increasingly widespread use of genomic testing, a growing number of these heterozygous carriers will be identified and require genetic counseling. In consequence, it is mandated that detailed phenotypic assessment should follow ambiguous genetic results. This is especially true, as there is emerging genotype-phenotype correlation data on heterozygous carriers of disease-causing variants in *COL4A3*/*COL4A4* [3, 37–39].

## Conclusion

In summary, this study shows that genetic reports, especially those dating back to pre-ACMG criteria times, need to be critically reevaluated on variant level but also genotype level giving the complexity of Alport spectrum disorder inheritance, particularly due to the variable and age-dependent expressivity.

## Supplementary information

Supplementary Table

## Data Availability

The data that support the findings of this study are available on request from the corresponding author.
